# Single‐cell biology: what does the future hold?

**DOI:** 10.15252/msb.202311799

**Published:** 2023-06-15

**Authors:** Maria Polychronidou, Jingyi Hou, M Madan Babu, Prisca Liberali, Ido Amit, Bart Deplancke, Galit Lahav, Shalev Itzkovitz, Matthias Mann, Julio Saez‐Rodriguez, Fabian Theis, Roland Eils

**Affiliations:** ^1^ EMBO Heidelberg Germany; ^2^ Center for Data Driven Discovery, Department of Structural Biology St Jude Children's Research Hospital Memphis TN USA; ^3^ Friedrich Miescher Institute for Biomedical Research (FMI) Basel Switzerland; ^4^ University of Basel Basel Switzerland; ^5^ Department of Systems Immunology Weizmann Institute of Science Rehovot Israel; ^6^ School of Life Sciences École Polytechnique Fédérale de Lausanne (EPFL) Lausanne Switzerland; ^7^ Department of Systems Biology Harvard Medical School Boston USA; ^8^ Department of Molecular Cell Biology Weizmann Institute of Science Rehovot Israel; ^9^ Max‐Planck Institute of Biochemistry Martinsried Germany; ^10^ Faculty of Medicine, Heidelberg University Hospital Heidelberg University Heidelberg Germany; ^11^ Helmholtz Munich Munich Germany; ^12^ Berlin Institute of Health at Charité Universitätsmedizin Berlin Berlin Germany

## Abstract

In this Editorial, our Chief Editor and members of our Advisory Editorial Board discuss recent breakthroughs, current challenges, and emerging opportunities in single‐cell biology and share their vision of “where the field is headed.”

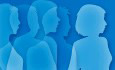

Single‐cell biology is a rapidly growing field that has made important contributions to our understanding of cell types, cell states, cell–cell interactions, and tissue architecture. Analyses at the single‐cell level are particularly well suited for dissecting dynamic biological processes that involve high cell–cell heterogeneity in a cell population, such as cell fate determination, tissue patterning, and development. Beyond these physiological contexts, single‐cell approaches have been extensively applied to study diseases, with cancer and infectious diseases being notable examples. Understanding cellular heterogeneity in disease contexts can have important clinical applications, including the development of improved diagnostic tools and targeted treatments. Furthermore, single‐cell biology enables cell reprogramming and engineering efforts for a variety of medical and synthetic biology applications.

Despite the numerous noteworthy contributions of single‐cell analyses, several questions remain open. What is the function of the various identified cell types and cell states? Are there more cell types and/or cell states that remain to be identified and how do they relate to the underlying dynamics of a given system? Cells do not function as single units; how can we understand the interplay between (bottom‐up) self‐organizing cell behaviors and top‐down constraints imposed by the microenvironment and a given physiological context? How can we infer rules and properties of cell–cell interactions from single‐cell data to better understand tissue organization? What kind of computational methods will enable integrating multiple data modalities and making new biological discoveries using the vast amounts of data that are continuously made publicly available? What new questions can be investigated by combining single‐cell approaches with upcoming developments in microscopy, imaging, and deep learning? The list goes on, and new questions as well as opportunities emerge as new technologies continue pushing the boundaries in the field.

In this Editorial, our Chief Editor and members of our Advisory Editorial Board discuss recent breakthroughs, current challenges, and emerging opportunities in single‐cell biology and share their vision of “where the field is headed.” Each of them gives a different perspective depending on their research focus, collectively offering an integrative view on the future of single‐cell biology. We are excited to share this Editorial and hope that it inspires discussions and new developments in the field.

## M Madan Babu: Integrative single‐cell biology for life, disease, and ecology

Cells are the fundamental units of life. While cell biology is an established field, recent technological advancements have significantly increased our capacity to obtain quantitative information about various cellular components, including transcripts, proteins, metabolites, and biomolecular structures. This surge in data throughput offers unprecedented opportunities to understand living systems, treat aberrant conditions, and engineer synthetic cellular systems.

During development, a single cell gives rise to a complex, multicellular organism. Even though all cells in an organism contain the same genetic material, they differentiate into distinct cell types, adopt different cell states, and acquire unique shapes. Investigating the interplay among diverse cell attributes, such as their spatial organization, migratory properties, physical connections, differentiation lineage, ultrastructure, and how these factors contribute to or disrupt homeostasis in various physiological and pathological contexts, is key for understanding multicellular systems. Exploring these questions will likely necessitate bridging multiple technologies, including cryo‐electron tomography, single‐cell spatial, genomic, and proteomic methods, large‐scale genetic perturbation approaches, as well as imaging techniques such as tissue clearing and expansion microscopy. Beyond the context of multicellular organisms, studies at the single‐cell level are well suited for understanding species diversity and interactions between different organisms in different ecological settings. For instance, in systems involving microorganisms, pathogens, and their interactions with host cells as well as their environment, single‐cell analyses can reveal molecular determinants (e.g., toxin–antitoxin systems) and principles governing interactions and emergent properties (e.g., cooperation, competition, persistence, and population dynamics).

The wealth of ever‐increasing data and breakthrough advancements in computational methodologies, such as deep learning techniques (e.g., transfer learning and large language models), will allow the standardization of disparate large‐scale datasets that are currently not easy to collate or mine. This will allow comparative studies across species, enabling molecular insights into adaptation to diverse environments, the identification of systems for investigating physiological and disease phenomena, and will facilitate drug discovery efforts in relevant model organisms. In the context of disease, these advances hold tremendous potential for understanding the impact of natural genomic variation, discovering the cellular loci of disease manifestation of pathological mutations and developing therapeutics. These new insights will empower us to engineer synthetic cellular systems for medical and biotechnological applications by fueling advances in cell reprogramming, cell‐based therapy, biosynthetic production of materials, and engineering microscale ecosystems within specific ecological niches.

To realize these possibilities, it is imperative to provide the next generation of scientists with the right education, training, scientific resources, and funding opportunities, enabling them to boldly pursue these challenging questions. Embracing the perspective that life on Earth constitutes an ecosystem of cells that interact with their environment and with each other will provide a conceptual framework for understanding life, treating human diseases, and preserving our ecosystem.

## Prisca Liberali: From single cells to tissue self‐organization

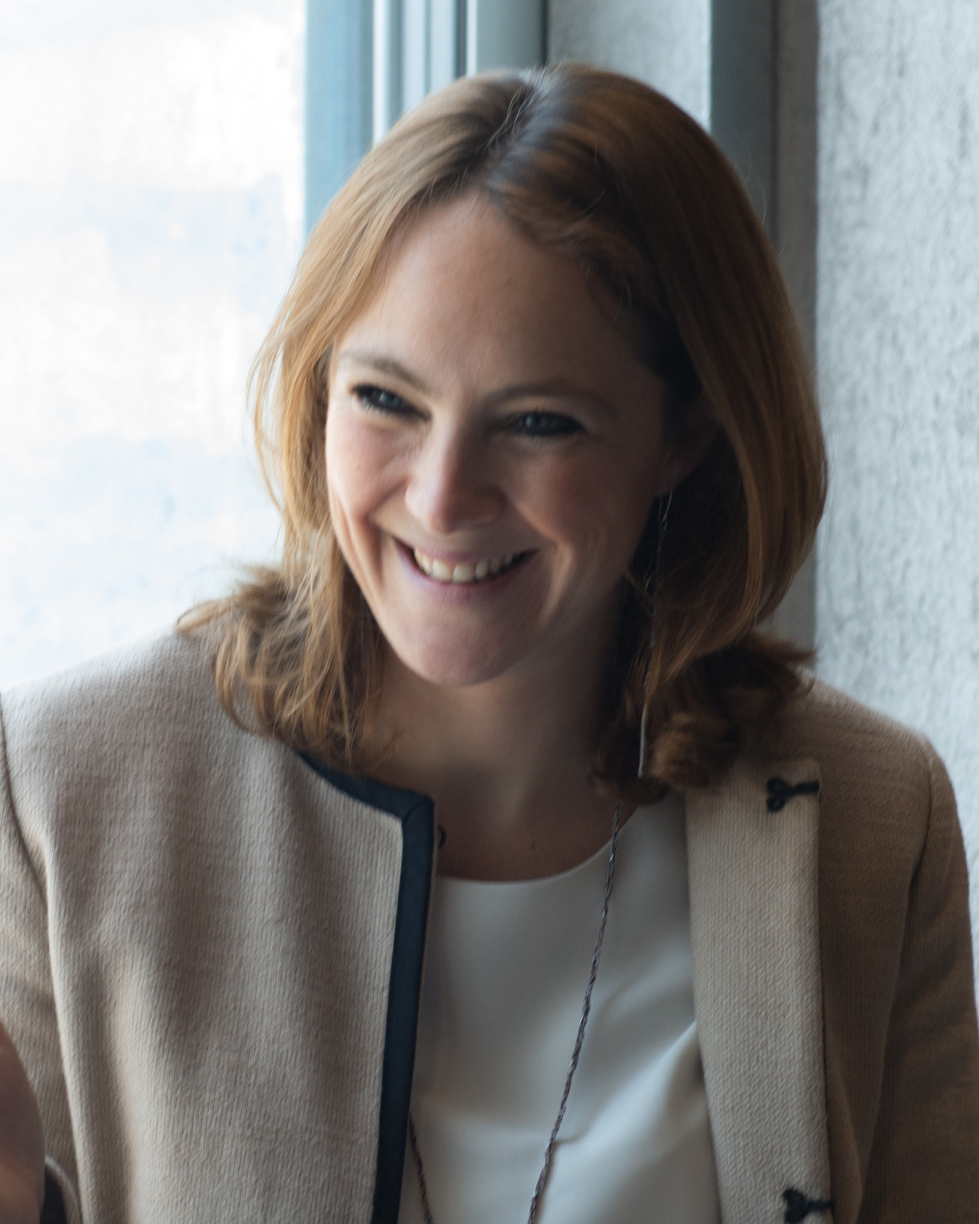

During development, in a series of spatiotemporal coordinated steps, individual cells differentiate into different cell types and establish tissue‐scale architectures and functions. Throughout life, continuous tissue renewal and regeneration also requires fine‐tuned spatiotemporal coordination of individual cells. How cellular interactions generate specific contexts and spatiotemporal coordination is a key question in biology. One main conceptual and technical problem is to understand the molecular and physical mechanisms that allow a cell, in a tissue, to sense its complex environment and to take individual but coordinated decisions. The field is moving fast in addressing these challenges by improving all single‐cell measurements: from transcripts and proteins to lipids. However, the integration of these measurements, especially as they cross biological scales, requires more conceptual understanding of self‐organization.

Indeed, some of the key challenges are that developmental and regenerative processes rely on intrinsic self‐organization of a population of cells and that tissues are not constructed machines, built top‐down, but they are built bottom‐up from individual cells that communicate with short‐ and long‐range cellular interactions. These interactions are not only chemical in nature but also mechanical and electrical. Therefore, individual cells need to have internal sensing mechanisms to know where and “when” they are, in order to coordinate their behavior in space and time, and control key process such as organ size and tissue homeostasis. We are now in very exciting times in which we can start integrating single‐cell measurements in highly accessible *in vitro* model systems, such as organoids, within mechanically controlled and engineered microenvironments, to understand how single cells have the intrinsic ability to generate emergent, self‐organized multicellular asymmetric systems.

## Ido Amit: Clinical applications of single‐cell technologies

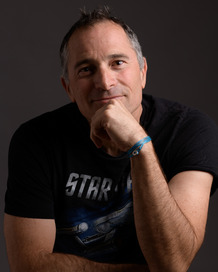

The field of single‐cell genomics has provided groundbreaking insights into diverse biological systems and helped identify new cell types and molecular pathways playing major roles in various physiological processes and pathologies. The single‐cell field is barely a decade old and is constantly evolving, driven by technological and analytical breakthroughs, such as the recent innovations in combining pooled CRISPR screens at single‐cell and genome‐wide resolution and other single‐cell multiomic methods, incorporating analysis of RNA along with proteomics, metabolomics, epigenetic data, and cell–cell signaling. Major near‐future stepping stones pushing the field forward are expected to be advancements in single‐cell spatial technologies, translating single‐cell data into a better understanding of cell–cell interactions and how different cells and cell types create diverse biological niches in the human body, and the incorporation of a temporal dimension into single‐cell biology. Additionally, integrating clinical data with single‐cell multiomics analysis is expected to provide novel insights into the underlying biology of many pathologies.

However, adding more layers of data is also creating challenges for the field. Integrating various types of data requires new computational tools and methods and is one of the major obstacles in the way toward the next level of single‐cell research. Such novel computational tools can enable faster incorporation of single‐cell technologies into clinical settings, which are currently hindered by the complexity of analysis required to decipher single‐cell data. Using these additional layers of single‐cell information and analysis methods can also shed new light on how the interactions between the immune system and tissue cells are involved in the emergence of various pathological conditions. This understanding can lead to the identification of signals that drive these pathologies and the development of novel groundbreaking therapies which are based on accurate modeling and understanding of current therapeutics and their vulnerabilities.

## Bart Deplancke: Single‐cell profiling of living cells

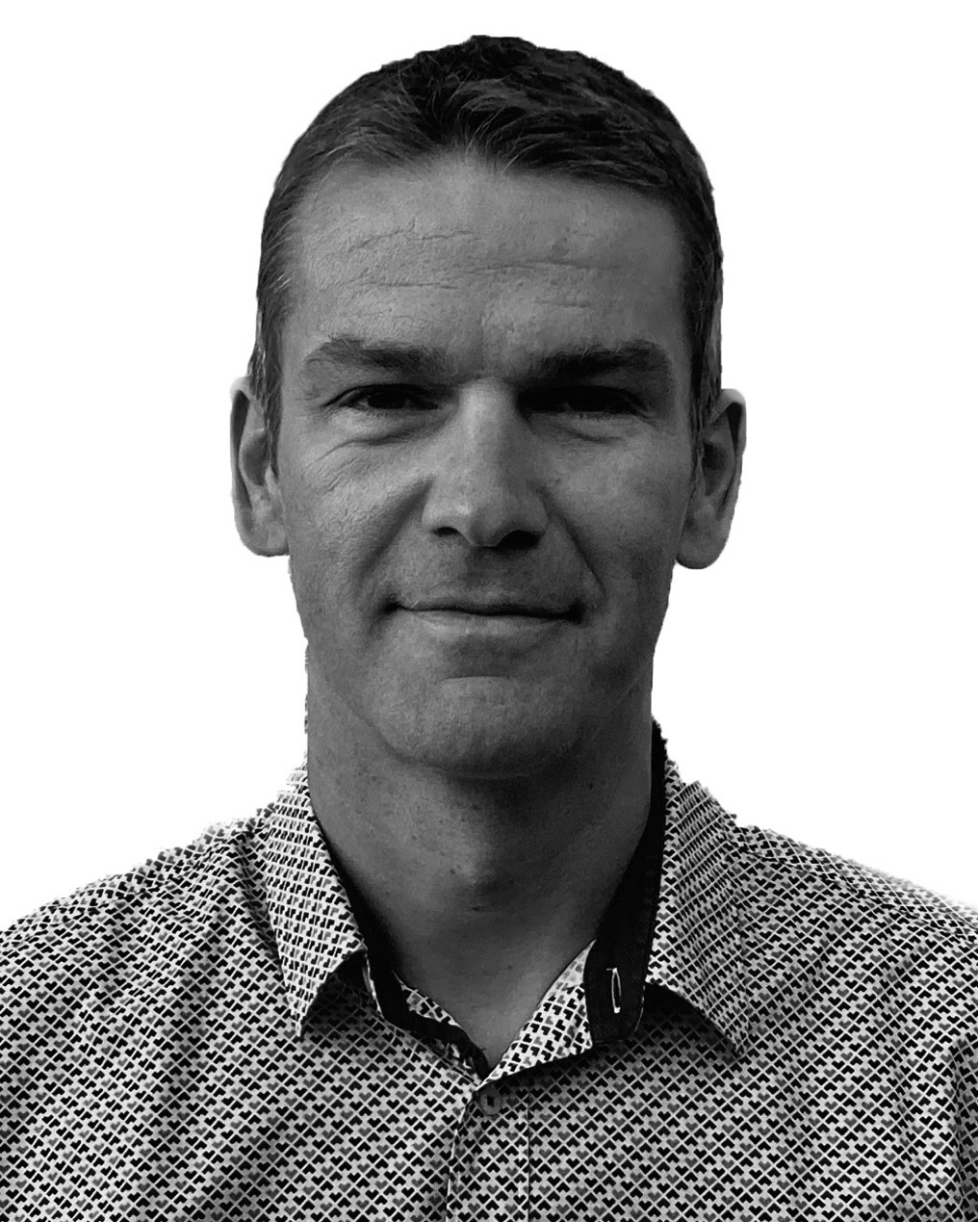

Our vision is that single‐cell analyses will increasingly cover distinct data modalities simultaneously, focusing not only on molecular profiling but also on phenotypically characterizing. A major limitation of current single‐cell omic profiling technologies in this regard is that they generally require sacrificing the analyzed cell, preventing downstream molecular or functional analyses on that same cell. To circumvent this issue, we developed Live‐seq, a method in which picoliter cytoplasmic biopsies are sampled from single cells and then processed by an ultrasensitive RNA‐seq protocol. Live‐seq provides unbiased gene expression profiles of single cells while keeping the cells alive and functional, unlocking exciting opportunities to study a range of biological processes, including stem cell differentiation, infection, disease progression, and drug resistance. Live‐seq enables both direct measurements of gene expression changes to reveal actual transition paths of single cells, as well as the recording of a cell's molecular history before a functional analysis. It thus provides new means to investigate cell state dynamics, and the reasons why cells that are subjected to the same endogenous or exogenous stimulation may respond differently. Moreover, since cell isolation is not required, individual cells can be targeted based on their relative spatial location. This offers a new way to investigate cell–cell interactions, for instance to study the response of noninfected bystander cells during infection, or stem cell signaling by proximal niche cells. While many applications are already within reach, future improvements to Live‐seq will include automating and multiplexing the sampling process to increase throughput, improving RNA‐seq sensitivity, and performing multiomics analyses. Enhancing throughput is essential for providing statistical robustness and the potential to explore rare cells. Increasing RNA‐seq sensitivity would improve the overall efficiency and allow using smaller‐volume biopsies per analysis, which in turn would make it possible to measure replicates of a single‐cell profile, or to perform multiple omics analyses of the same cell, since the cytoplasmic biopsies contain not only the transcripts but also proteins and metabolites. We anticipate that an increased sensitivity will also strongly benefit alternative live‐cell sampling approaches such as glass nanopipettes and nanostraws. The latter have already been used to collect single‐cell biopsies for molecular analyses and have the potential to further democratize live‐cell omic profiling and phenotyping.

## Galit Lahav: Bridging dynamical systems with single‐cell omics

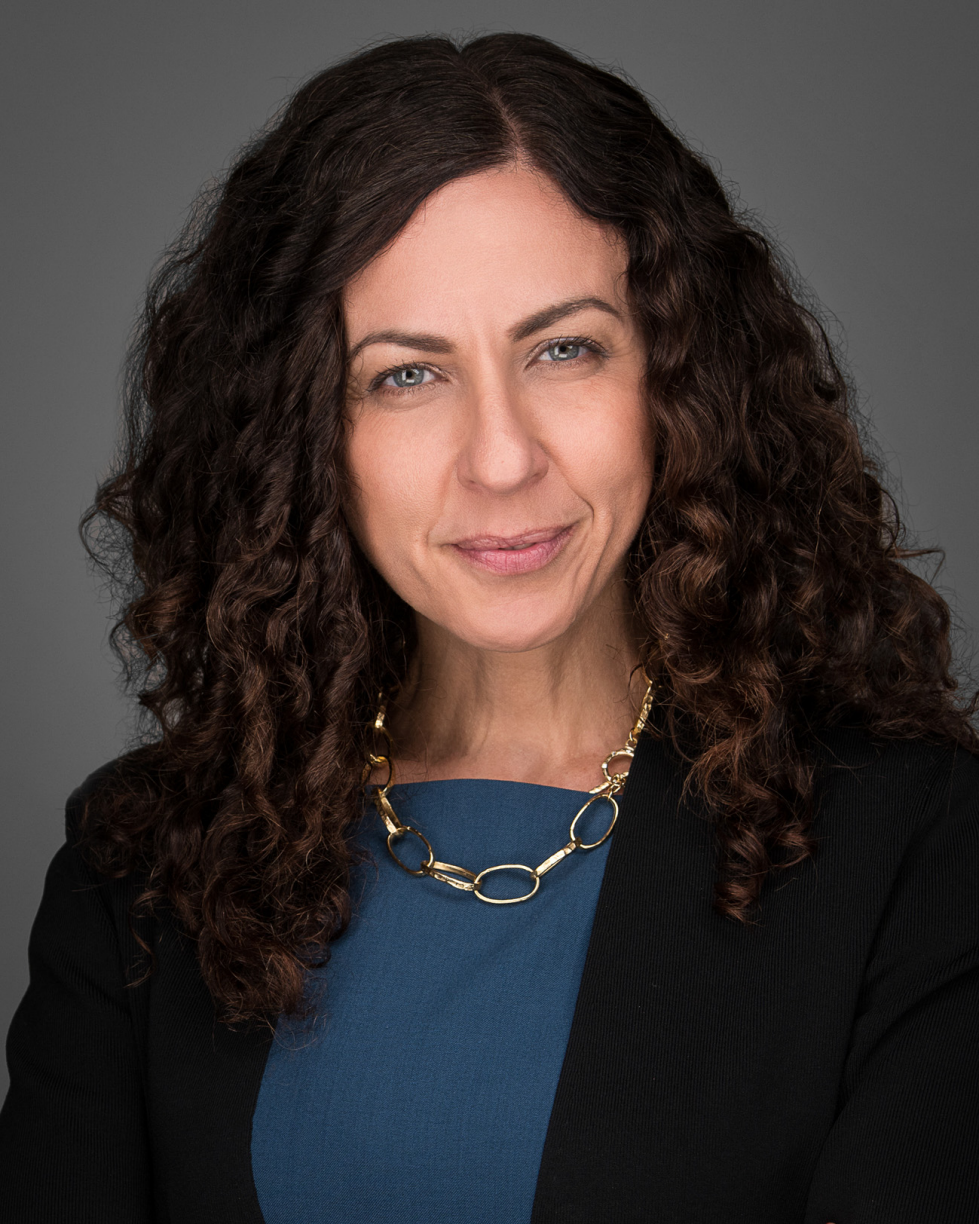

The field of single‐cell biology is rapidly evolving and has already impacted a wide range of biological and biomedical research areas. For this Editorial, I will share what I envision as one of the next major needs in studying biology in single cells. Single‐cell studies are generally pursued using two main approaches. One approach focuses on measuring the dynamics of one or a few signaling molecules using biosensors and imaging of live cells in response to specific stimuli and connecting them with phenotypic outcomes. Recent examples for this approach include studying the dynamics of p53, NFkB and phospho‐ERK and associating specific features of their dynamics with the expression of their target genes and cellular behaviors (e.g., proliferation, cell death, and differentiation). The second approach is focused on measuring the levels of signaling molecules globally in a large number of fixed single cells. Recent examples for this approach include single‐cell RNA‐seq measurements to map the gene expression changes during early embryonic development and revealing the heterogeneity of T cells in response to infection. A robust high‐throughput platform that integrates these two approaches over long periods of time is needed but still missing. Connecting real‐time measurements of signaling molecules in live cells with single‐cell omics is critical for the development of powerful predictive models of how biology works, how disease emerges and the outcomes of single cells to therapy. In addition, since measuring dynamics in the clinic is not possible, associating dynamics with omics data and cellular outcomes is also promising for identifying new disease biomarkers.

## Shalev Itzkovitz: Understanding the principles of tissue organization

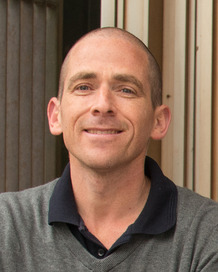

Solid tissues are often composed of repeating anatomical structures, such as the intestinal villi, the liver lobules, and the kidney nephrons. These structures are polarized due to blood flow or morphogens. As a result, cells sense unique microenvironments with varying levels of oxygen, nutrients, microbial content, and other factors depending on their location within the structure. As an evolutionary adaptation, tissues assign different tasks to cells at different zones, a phenomenon termed “zonation.” Single‐cell technologies have contributed immensely to our understanding of zonation patterns across tissues. Comprehensive zonation maps were attained by breaking tissues apart and measuring the complete transcriptomes of thousands of cells, while in parallel quantitatively mapping the spatial expression patterns of representative “landmark genes” to infer the zone of origin of the dissociated cells. Spatial transcriptomics further facilitated the reconstruction of global spatial gene expression patterns *in situ*. An outstanding challenge in tissue biology is the reconstruction of spatial expression patterns of thin elongated cells, such as fibroblasts, endothelial cells, and neurons, which often exhibit intracellular RNA polarization. An example is the intestinal telocyte, a niche cell that can span hundreds of microns in length and that localizes distinct mRNA molecules to distinct coordinates *within* the cell. This ability to localize mRNAs in distinct cellular compartments over hundreds of microns defies our basic definition of cells as the fundamental building blocks of a tissue. The steadily increasing throughput and spatial resolution of spatial transcriptomics techniques can expose the spatial expression programs of such challenging cell types.

## Matthias Mann: Single‐cell proteomics

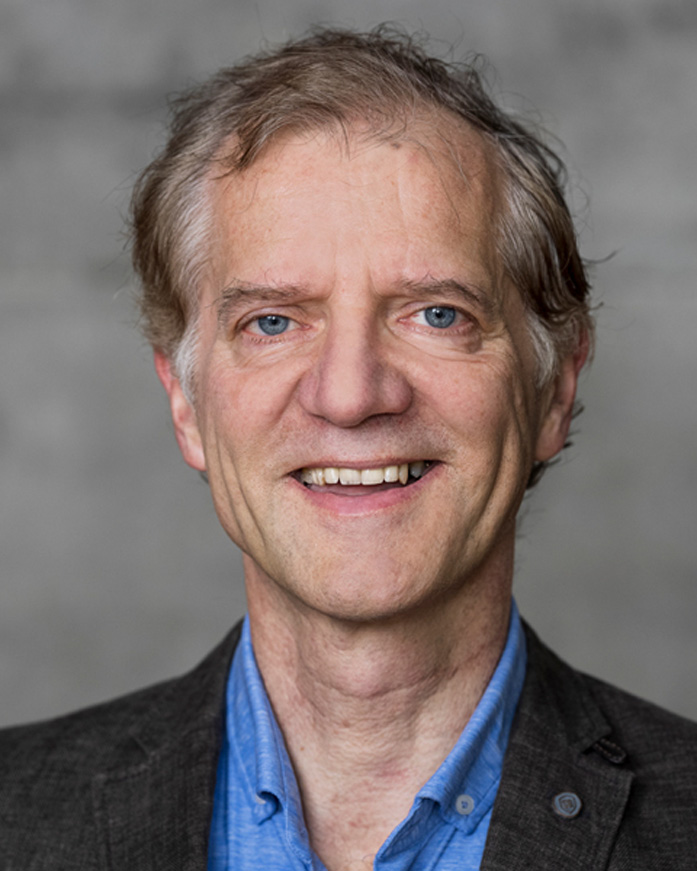

Single‐cell proteomics (scProteomics) is an emerging technology at the heels of scRNA‐seq, which has revolutionized our understanding of cell types and cell–cell heterogeneity. scProteomics has been made possible by great advances in sample preparation methods, the mass spectrometers as well as scan modes and data processing algorithms. Together they enable identification and quantification of thousands of proteins per cell. Rather than repeat analyses similar to scRNA‐seq, which were mainly done on dissociated cells, our group proposes to analyze single cells or cell types directly from fixed tissue after AI‐guided laser microdissection. This “Deep Visual Proteomics” provides a rich spatial context to the proteomics results and captures biological variability while averaging out the random variability in populations with the same functional attributes. In the next few years, scProteomics is expected to continue to advance in sensitivity, throughput, and spatial resolution. The ability to read out the functional effects of biological heterogeneity will provide new insights into the biology of individual cells. scProteomics may become particularly valuable in cancer diagnostics, where it can uncover cellular heterogeneity and the functional roles of stem cell niches. Deep Visual Proteomics in particular is well suited for this task, as it can uncover biologically meaningful differences that are hidden in bulk data and can bypass some of the current technological limitations of scProteomics such as throughput and depth of coverage. Clearly, scProteomics is a powerful tool for understanding cellular biology and has the potential to revolutionize our understanding of disease at the single‐cell level.

## Julio Saez‐Rodriguez: Integrating multiomics at the single‐cell level

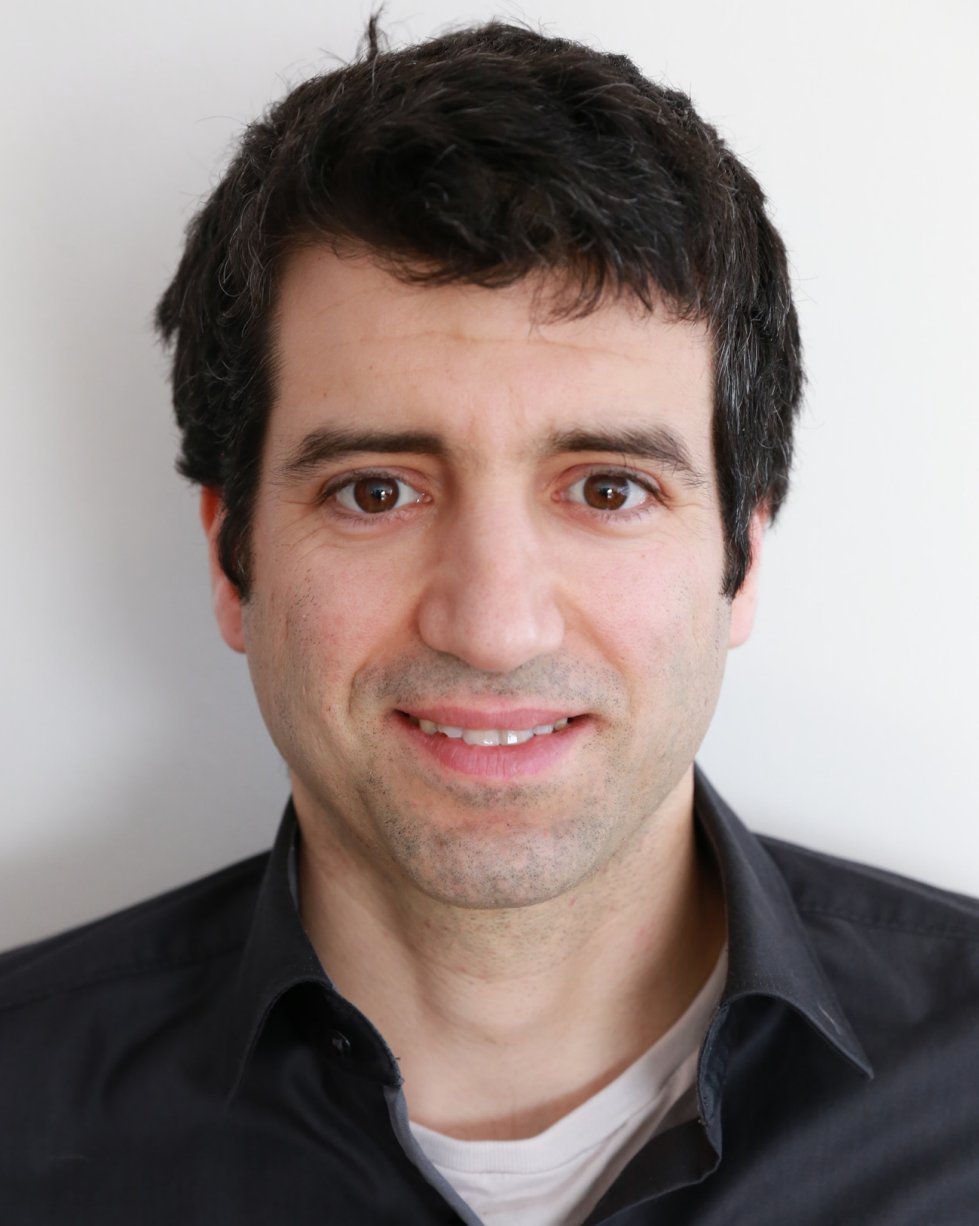

Single‐cell and spatially resolved multiomic technologies allow us to “see” fundamental principles of multicellular systems that are impossible to access using bulk technologies. The combination of different omics layers provides complementary information on the diverse molecular processes in the cell. We expect that these data modalities, along with the corresponding statistical and machine learning methods, will be instrumental for studying the coordinated functions of cells in tissues and organs. Specifically, when used in large cohorts of patients, these strategies will inform us of the variability in tissue structure and thereby the misregulated multicellular programs that drive disease phenotype.

The scope and resolution of the data, in particular when coupled with perturbation experiments, will allow us to learn more about causality in cellular phenotypes. For this, we believe that machine learning has to be supported by our—admittedly incomplete and unbiased—prior knowledge of the underlying molecular mechanisms. These insights can also serve as the foundation for dynamic mechanistic models that will eventually allow us to simulate the effect of drugs or other perturbations in tissues. All these advances should ultimately improve the prognosis of disease progression and the treatment of patients.

## Fabian Theis: Toward a foundational machine learning model of the cell

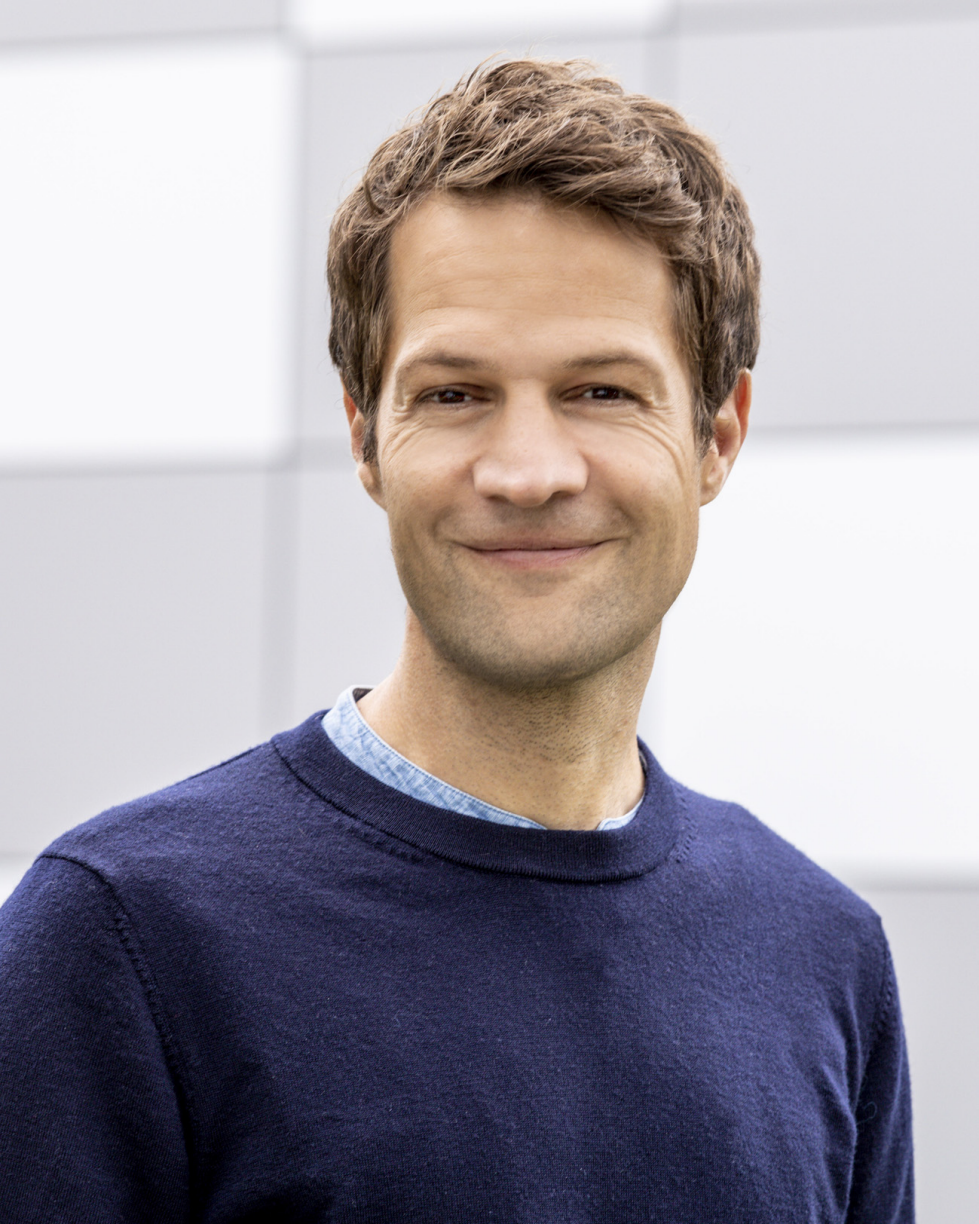

Single‐cell biology first emerged using microscopy, and it has since come a long way. The scale has expanded dramatically, in terms of both throughput and the number of measured state variables and modalities. It therefore comes as no surprise that modern data science methods have become key for the analysis of the resulting big datasets, with machine learning and artificial intelligence (AI) being the key driving methods for, for example, identifying cellular states and cell types, learning about single‐cell dynamics or integrating data across conditions, perturbations, and scales. The next big thing in AI is large language models, or more generally called *foundation models*, defined as large generative neural networks trained on vast datasets in an unsupervised fashion, thereby enabling many downstream applications in parallel mode that learns domain concepts across tasks. I am looking forward to seeing these ideas being applied in scientific AI in general and in single‐cell biology in particular.

The single‐cell biology field has been dominated by unsupervised machine learning approaches, from early clustering and pseudotime methods to cohort integration or perturbation models. Recently, similarly to what has happened in computer vision a decade ago, deep learning has started to outperform classical machine learning for sufficiently complex tasks. The natural next step will be to integrate samples not only within a modality, perturbation, organ, or even species, but instead aim at learning cellular variation across all available covariates. The resulting deep embeddings will form an early version of a *foundation model of the cell* that will not only connect molecular but also spatial and morphometric modalities in a generative fashion. Once such models become robust and more broadly available, many questions that we now approach with separate algorithms could be tackled using this foundation model. This model would be able to rapidly contextualize new samples or predict the effect of unseen perturbations and enable the design of such perturbations toward desired states, for example, in the case of particular disease states. Similarly, comparisons across organs—desirable, for example, within an immune cell atlas—could be made efficiently as soon as cells are embeddable into a joint latent space or model. Developmental or evolutionary questions could be approached by observing cellular state changes across time or species in such an integrated model.

As modern language models enable text generation as answer to a query, we will be able to query a cellular foundation model about unseen, new cell states across health and disease.

## Roland Eils: Making the most out of single‐cell data

**Figure:**
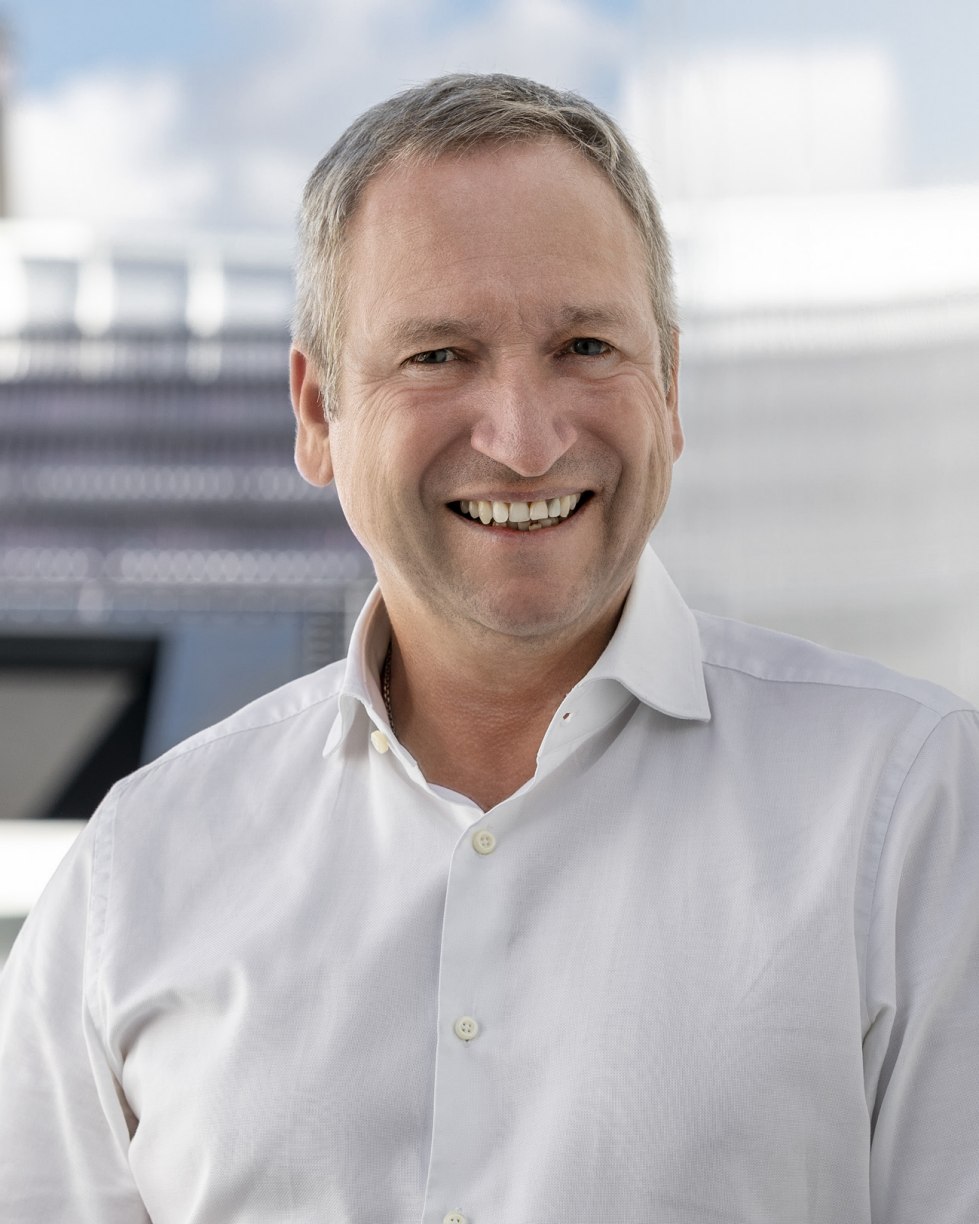
Copyright Sebastian Runge

The relatively young field of single‐cell biology has been developing rapidly. While standard 3’ mRNA sequencing has become commonplace across all areas of biomedicine, single‐cell biology is now becoming increasingly multimodal and multiscale. This presents significant challenges for analyzing the increasingly complex datasets. Integrating multimodal data and analyzing and interpreting spatial single‐cell sequencing data are not straightforward tasks. Computational pipelines for data integration and analysis are not standardized, and they vary widely from laboratory to laboratory, making it difficult to compare results even within the same biomedical field.

International initiatives, such as the Human Cell Atlas initiative, are striving to establish standardized procedures for data analysis and interpretation. Standardized cell‐type signatures are urgently needed, but it is unclear how best to achieve this. Furthermore, pipelines and signatures will change over time, necessitating their versioning. Achieving consensus on these issues will have a significant impact on the field and will help propel single‐cell biology to the next level.

There are numerous exciting applications for single‐cell biology across the life sciences. For example, understanding the complex spatial organization and regulation of tumors in different microenvironments, in conjunction with immune cell phenotyping using techniques such as T‐cell receptor sequencing, will almost certainly have a huge impact on cancer diagnostics and the design of entirely novel therapies. In the long term, a deeper understanding of disease onset and progression based on multiscale and multimodal single‐cell sequencing will help to effectively treat and potentially even cure diseases which currently have a very bad prognosis.

